# Design of a CMOS Image Sensor with Bi-Directional Gamma-Corrected Digital-Correlated Double Sampling

**DOI:** 10.3390/s23021031

**Published:** 2023-01-16

**Authors:** Jaehee Cho, Hyunseon Choo, Suhyeon Lee, Seungju Yoon, Gyuwon Kam, Sooyoun Kim

**Affiliations:** Department of Semiconductor Science, Dongguk University, Seoul 04620, Republic of Korea

**Keywords:** CMOS image sensor, dynamic range, gamma correction, log-exponential counter, single-slope analog-to-digital converter

## Abstract

We present a 640 × 480 CMOS image sensor (CIS) with in-circuit bi-directional gamma correction with a proposed digital-correlated double sampling (CDS) structure. To operate the gamma correction in the CIS, the transfer function of the analog-to-digital converter can be changed by controlling the clock frequency of the counter using analog CDS. However, the analog CDS is vulnerable to capacitor mismatch, clock feedthrough, etc. Therefore, we propose a digital-CDS method with a hold-and-go counter structure to operate the bi-directional gamma correction in the CIS. The proposed CIS achieves a 10-bit resolution using a global log-exponential counter and configurable column reset counter with a resolution of 8/9 bits. The sensor was fabricated in a 0.11 μm CIS process, and the full chip area was 5.9 mm × 5.24 mm. The measurement results showed a maximum SNR improvement of 10.41% with the proposed bi-directional gamma-corrected digital-CDS with the hold-and-go counter. The total power consumption was 6.3 mW at a rate of 16.6 frames per second with analog, pixel, and digital supply voltages of 3.3 V, 3.3 V, and 1.5 V, respectively.

## 1. Introduction

**Figure 1:**
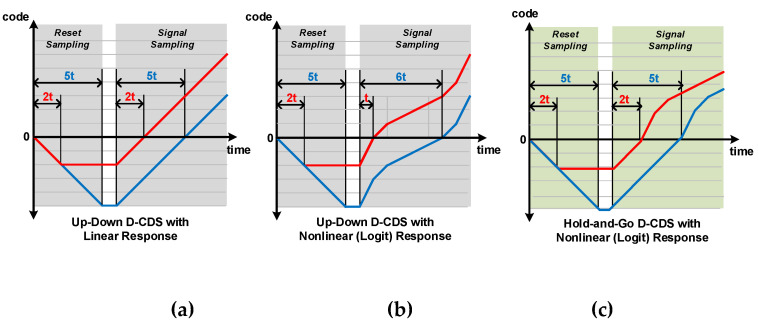
D-CDS output using up-down counter with (**a**) linear response and (**b**) nonlinear response, and (**c**) using hold-and-go counter with nonlinear response.

Wide-dynamic-range (WDR) CMOS image sensors (CISs) have been developed as the demand for surveillance cameras, automobiles, and medical cameras has increased. Several techniques to obtain a wider dynamic range image at the circuit-algorithm level have been proposed [1,2]. Additionally, several techniques to achieve WDR without changing the pixel structure have been presented [3,4,5]. By adjusting the analog/digital gains, in-circuit gamma correction techniques can enhance image quality [6,7,8]. In general, gamma correction is performed in the image signal processor (ISP) to compensate for the limited DR of displays. Instead of memory and the complex digital circuits required for the gamma correction in ISP, the visibility of images can be improved by increasing the gain in low-light areas by adjusting the slope of the ramp generator and the frequency of the digital counter in CIS. Applying conventional gamma curvature using a digital counter has been suggested [7,8]. However, conventional gamma correction can lead to data loss after correction if the original image contains a highly illuminated, saturated area. To solve this problem, bi-directional gamma correction, which can correct both dark and bright areas simultaneously, was implemented with a logarithmic-exponential counter (LEC) and analog-correlated double sampling (A-CDS) [9]. However, the A-CDS technique can be affected by noise from capacitance mismatch caused by process variation or clock feedthrough from the switch as the signal toggles. Additionally, as higher resolution is in demand these days, the noise sources from A-CDS can affect overall image quality. Thus, we considered the implementation of digital CDS (D-CDS), which is less affected by noise from process variation and signal transition. D-CDS can be achieved using a conventional up-down (UD) counter. However, the D-CDS implemented with a conventional UD counter operates through the linear in-output response, as shown in Figure 1a. Reset voltages, including an offset voltage, are each sampled after 2 t and 5 t. After 2 t and 5 t from the moment signal voltage sampling starts, the code becomes ‘0′, in which CDS operation is performed. However, bi-directional gamma curvature applies different gains at different luminance areas. Thus, it has a nonlinear in-output response. If bi-directional gamma correction is applied using a UD counter, the power consumption and area considerably increase, and a CDS operation error occurs. When the signal voltages are the same while the reset voltage varies, as depicted in Figure 1b, a nonlinear response causes an error that results in different timing of outputting the 0 code (t, 6 t). To remove the error generated while implementing the nonlinear response in the UD counter, reset and signal sampling should be performed separately within the column, so the counter area per column is twice as large as the linear response in the UD counter.

Thus, the hold-and-go (HG) technique [10,11], which performs the D-CDS operation using both global and column counters, is used to achieve bi-directional gamma correction, as shown in Figure 1c. In the case of the HG counter, the column counter operates only when the reset voltage is sampled, and the global counter operates when the signal voltage is sampled. Therefore, in the case of a 10-bit ADC, only an 8-bit or 9-bit column counter (8/9-bit column counter in this paper) for reset sampling is required. For the column-parallel ADC in CIS, a small area of the column circuit is required to obtain the small total area. When using a 9-bit reset counter, the column counter area is reduced by about 10%. On the other hand, since a 20-bit column counter is required to obtain a 10-bit non-linear response with the UD counter, the column counter area can be reduced by 55% using only a 9-bit column counter with the proposed HG D-CDS, as shown in Figure 1c. Therefore, by implementing D-CDS using the HG method, the image sensor’s overall power consumption and area are less than those of conventional CIS with the UD counter.

## 2. Proposed CIS Structure

**Figure 2:**
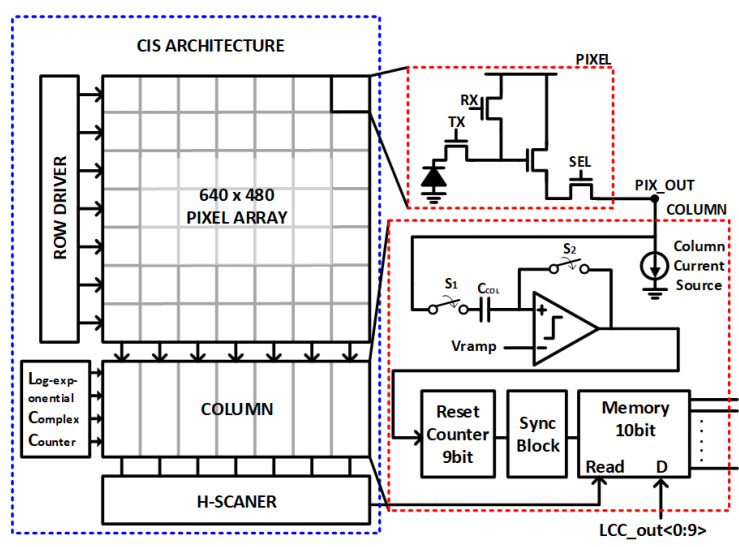
Full block diagram of the proposed CIS.

The proposed CIS has a VGA resolution of 640 × 480 pixels and a 10-bit column-parallel single-slope analog-to-digital converter (SS-ADC) structure using the hold-and-go (HG) technique, as depicted in Figure 2. The prototype sensor consists of a pixel array, a 9-bit column counter for digital-correlated double sampling (D-CDS), a logarithmic-exponential counter (LEC) for bi-directional gamma correction as a global counter, and other digital periphery blocks. Additionally, the analog circuits of each column consist of the comparator, a 10-bit 2-stage static random-access memory (SRAM), and a column decoder for H-scanning. Two-stage SRAM enables outputting values at the second stage while getting simultaneous input into the first stage. The LEC has five operation modes as follows: linear mode, gamma mode, ½-point mode (M), ¼-point mode (L), and ¾-point mode (H).

### 2.1. Correlated Double Sampling (CDS)

In CIS, the Pixel Array consists of numerous pixels. These pixels have different characteristics for each pixel due to errors in the process. Even if the same light is irradiated to all pixels due to different threshold voltages of transistors in each pixel, each pixel output has different offset voltages. These different voltages result in fixed-pattern noise (FPN) that can be removed by CDS. The pixel outputs two voltages: a reset voltage and a signal voltage caused according to the intensity of light, and the difference between the two voltages is the input voltage range of the SS-ADC (=0.8 V in this paper). Since these two voltages come through the same pixel, they have a constant offset error. The CDS technique is used to remove the offset error by sampling both the pixel output during the reset process and pixel output by light and finding the difference between the two signals. The types of CDS techniques are divided into Analog-CDS using a capacitor and circuit feedback and global counter and Digital-CDS subtracting the digital code of the reset voltage and the digital code of the signal by light using a separate memory or a counter inside the column.

**Figure 3:**
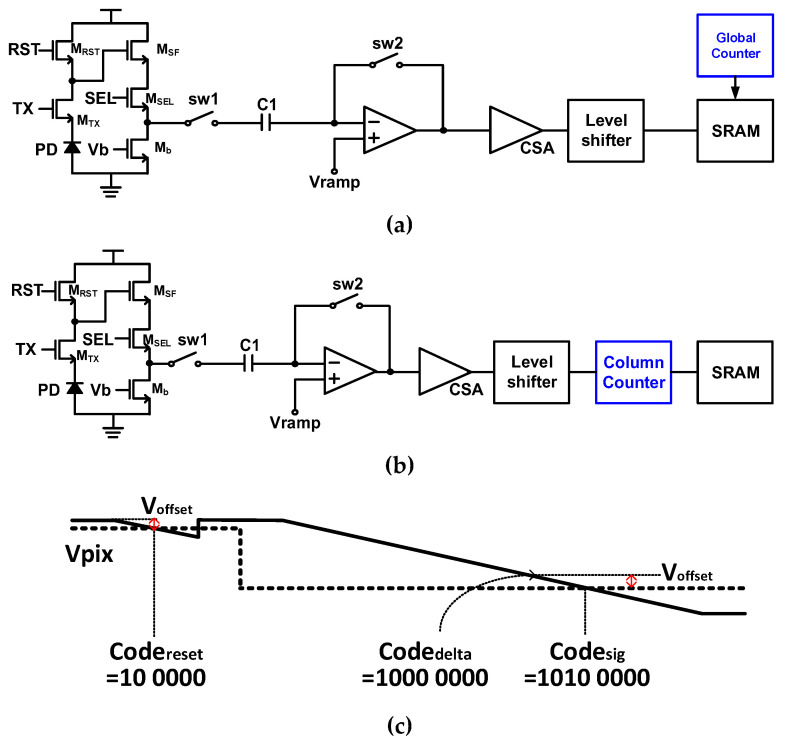
The block diagrams of (**a**) A-CDS and (**b**) D-CDS, and (**c**) Operation of D-CDS.

Figure 3a shows the block diagram of A-CDS that performs CDS using feedback using a switch (sw2) and capacitor (C1). According to the law of conservation of charge, the difference between the reset voltage and signal voltage can be obtained. Such A-CDS can be operated relatively simply, but has a disadvantage in that accuracy is lower than that of D-CDS due to mismatch in the capacitor process, kT/C noise, errors caused by clock feedthrough, and charge injection of switches. Figure 3b,c show the structure and operation principle of D-CDS and has a counter in each column. As shown in Figure 3c, first, the reset voltage is sampled and converted, and then the value is subtracted from the code obtained by converting the signal voltage to obtain the difference between the two signals with the offset error removed. As shown in Figure 3c, code_delta_ is the value obtained by subtracting code_reset_ from code_sig_, confirming that Voffset is removed.

### 2.2. D-CDS Operation Using Hold-and-Go Counter Structure

**Figure 4:**
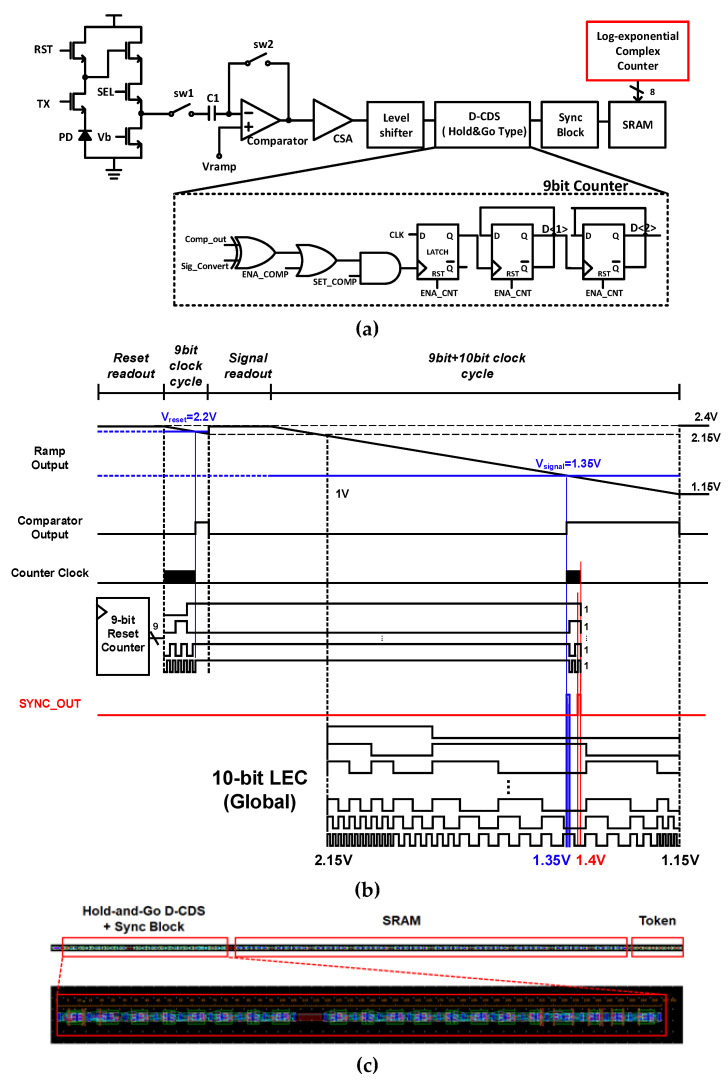
(**a**) Block diagram and (**b**) Timing diagram of SS-ADC with D-CDS using the HG Counter, and (**c**) 1-column layout of the HG counter and SRAMs.

Figure 4a,b show the block diagram of D-CDS with the HG method in SS-ADC and the principle of the D-CDS operation using the HG method. The reset counter (RC) starts counting when the clock cycle for the 512 code (9 bit) starts. The RC holds its output when the comparator output is flipped from ‘L’ to ‘H’ by the reset voltage. After the 9-bit clock cycle, the 9-bit + 10-bit clock cycle starts for signal voltage sampling. When the comparator output turns into ‘H’ from ‘L’ by the signal voltage, the RC counts again from the held code until it reaches the maximum code. When the RC outputs the maximum code, MSB, which is the input of the sync block, turns into ‘H’. At the sync block, a sync signal is generated when the RC reaches the maximum code 111111111(2). Additionally, for energy efficiency, a configurable structure that can achieve both an 8-bit and a 9-bit resolution RC was applied. Figure 4c shows a 1-column digital block layout. The total height is 840 μm, of which the height of HG D-CDS is 228 μm. The total height of 1-column SS-ADC is 998 μm.

### 2.3. Log-Exponential Counter

**Figure 5:**
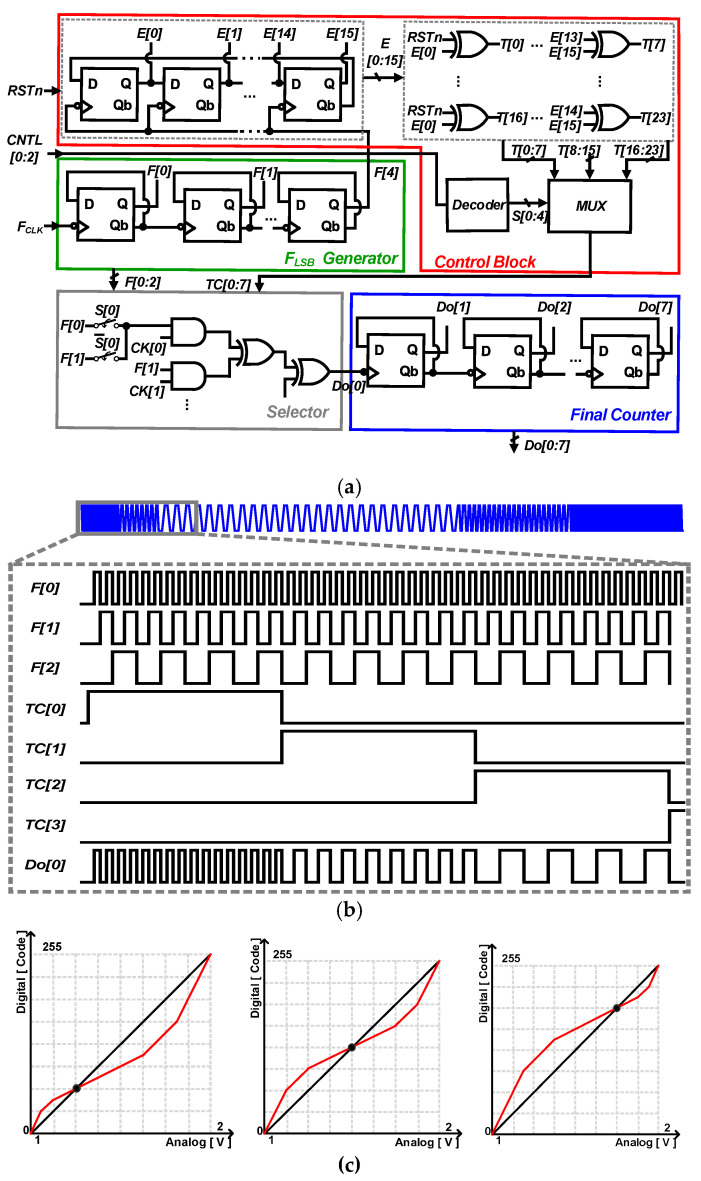
(**a**) Block diagram of the global LCE, (**b**) timing diagram, and (**c**) different bi-directional gamma curvatures.

Figure 5a,b shows the block diagram and timing diagram of the global LEC consisting of an F_LSB_ generator, control block, selector, and final counter. The F_LSB_ generator consists of five Flip-Flops to generate F<0:2>, which determines the digital gain with three different frequencies. The control block generates TC<0:7>, which determines the intervals at which the digital gains are applied. The selector generates the Final LSB signal Do<0> by applying F<0:2> to each interval made by the control block. Each operation mode can be selected according to the 3-bit control signal CNTL [0:2]. The final counter generates a 10-bit final output from Do<0>. Figure 5c shows the bi-directional gamma curvature applied in the LEC. Each curvature illuminance area to be corrected is selectable.

## 3. Experiment Results

**Figure 6:**
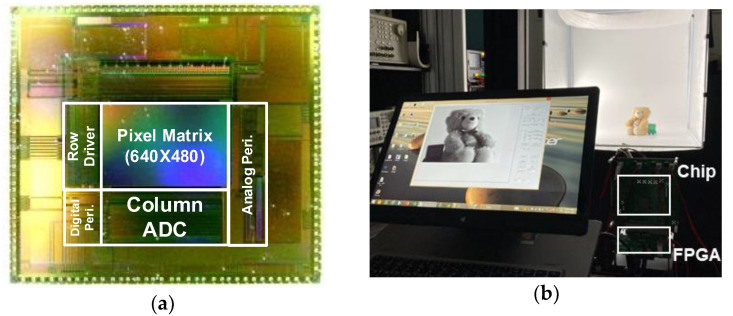
(**a**) Chip microphotograph and (**b**) test environment.

**Figure 7:**
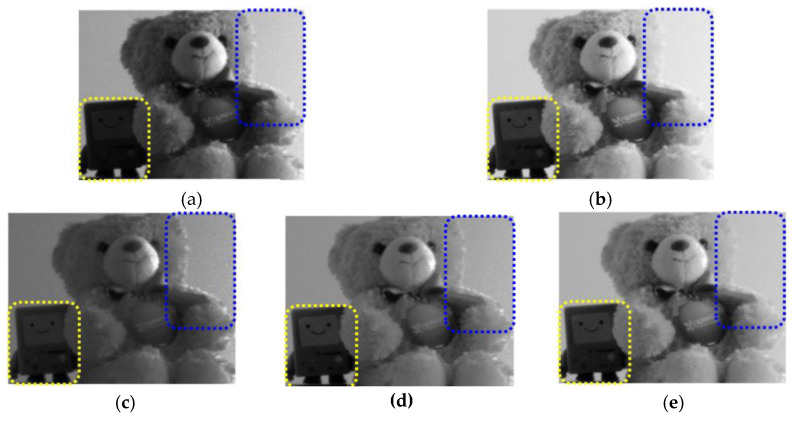
Evaluated images @ 550 lux. (**a**) Normal image with linear mode; (**b**) gamma-corrected image; bi-directional gamma-corrected images for (**c**) ¼ pt., (**d**) ½ pt., and (**e**) ¾ pt.

The prototype of the proposed CIS was fabricated with the 0.11 μm CIS process. Figure 6a shows the chip microphotograph of the prototype CIS with an area of 5.9 mm × 5.24 mm in the full chip. To proceed with the images captured with the proposed CIS, the measurement environment is shown in Figure 6b, and the measurement sequence was as follows. An FPGA board, XEM3050 (Xilinx Spartan-3 FPGA Integration Module) was used to check the control signal application and image output for driving the image sensor on a computer. Through the ISE program, the control signal was applied to the designated pin of the image sensor with Verilog, and the Verilog-A coding was completed to sequentially read the output of the sensor and display it on the computer’s image viewer. Using the Opal Kelly board commercially available from Xilinx, the FPGA was driven based on the USB interface, and the measurement was conducted by checking the final image displayed in the image viewer. Figure 7 shows the captured images from the proposed CIS. The bottom-left side of Figure 7a captured in linear mode is dark and invisible. As depicted in Figure 7b, in the image corrected with the gamma curvature, the dark area becomes more visible, revealing the facial expression of the object, but the visibility of the upper-right side of the object is poor. The image with the proper bi-directional gamma curvature (Figure 7d) shows enhanced visibility in the low-illumination area, while the object is distinguishable from the background in the high-illumination area.

### 3.1. Modified-Absolute Mean Brightness Error

DR represents the ratio of the maximum output signal level to the noise floor level and depends on the characteristics of the pixels. Therefore, gamma correction techniques in ISP and circuits in CIS do not improve DR, and the measured DRs with different bi-directional gamma modes are identical [9]. However, as shown in Figure 7, the visibility of images can be enhanced with the bi-directional gamma correction technique. Since image quality assessment can be influenced by subjective opinion, an objective evaluation concept was suggested. The absolute mean brightness error (AMBE) refers to the absolute error value between the original image and the corrected image [12]. The AMBE is expressed in the equations below. When *E[i]* and *A(i,j)* are defined as Equation (1), the mean gray level *E[X]* of the image consisting of m × n array is defined as Equation (2).

(1) $$E\left[i\right]=\frac{\mathrm{Number}\;\mathrm{of}\;\mathrm{occurences}\;\mathrm{of}\;\mathrm{the}\;\mathrm{intensity}\;\mathrm{level}\;i}{\mathrm{Number}\;\mathrm{of}\;\mathrm{intensity}\;\mathrm{levels}}$$

(2) $$E\left[X\right]=\frac{1}{m\times n}\sum_{i=0}^{m-1}\sum_{j=0}^{n-1}A\left(i,j\right)\;\mathrm{where}\;A(i,j)=\mathrm{Intensity}\;\mathrm{level}\;\mathrm{of}\;\mathrm{pixel}\;A\;\mathrm{at}\;\mathrm{row}=i,\;\mathrm{col}=j.$$

The AMBE can now be defined as Equation (3).

(3) AMBE=|E[Y]−E[X]|

(E[X]=Mean gray level of the original image,E[Y]=Mean gray level of enhanced image).

**Figure 8:**
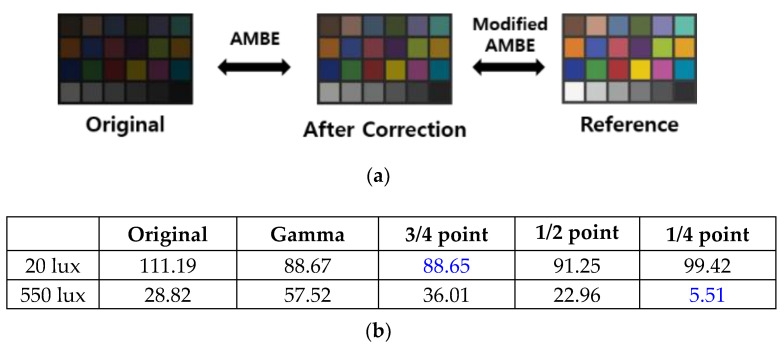

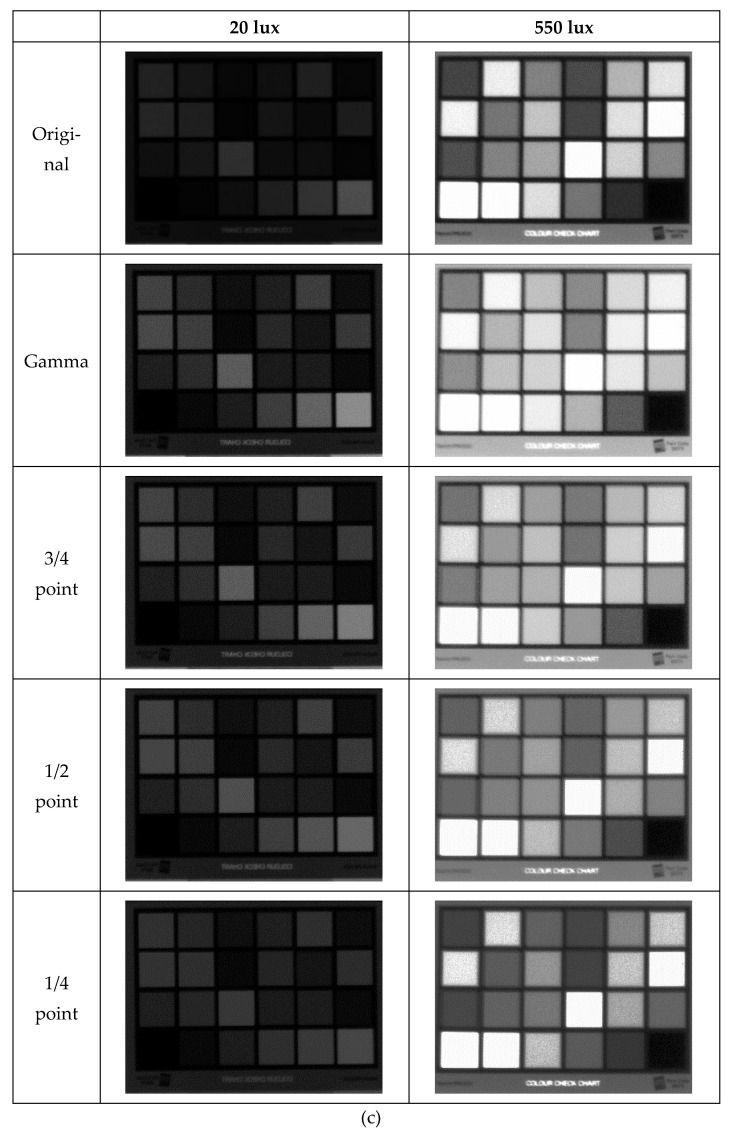
(**a**) AMBE and M-AMBE concepts; (**b**) calculated M-AMBE; and (**c**) captured images.

Since the output of the LEC is a result of bi-directional gamma correction, a modified-AMBE (M-AMBE) was used for comparison. The M-AMBE refers to the mean brightness error between the reference and the corrected image, as shown in Figure 8a. As the color blocks inside the Macbeth ColorChecker have solid values, each color block was converted into standard RGB (sRGB) values and then converted into grayscale values. The M-AMBE was compared using the grayscale values. As shown in Figure 8b,c, the ColorChecker image captured at low illumination (20 lux) with a 3/4 point bi-directional gamma correction showed a maximum M-AMBE improvement of about 20% compared to the linear mode counter output. In the case of high illumination (550 lux), the ColorChecker image captured with the ¼-point bi-directional gamma correction showed the best M-AMBE characteristics.

### 3.2. Signal-to-Noise Ratio

**Figure 9:**
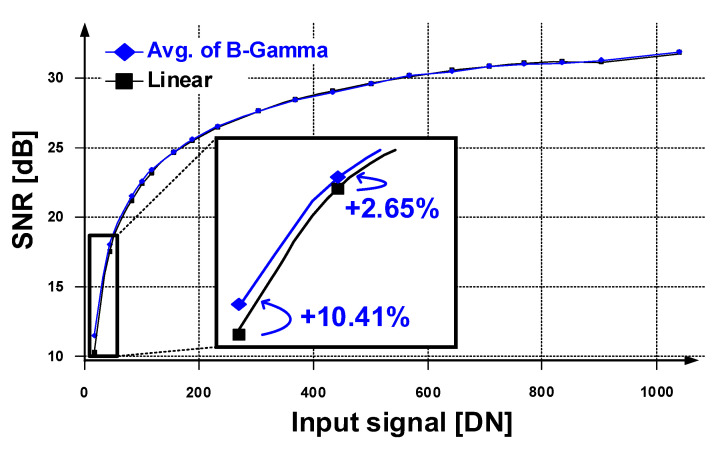
Measured SNR graph of the prototype CIS.

Table 1 shows the measured signal-to-noise ratio (SNR) with different response modes of the prototype sensor. At the low illumination of the code in the digital number (DN) range between 0 to 20, the SNR of all bi-directional gamma modes is higher than that of the linear mode due to 2 × higher clock frequency that works like multisampling. As shown in Figure 9, the SNR improves at low illumination, but as the illumination increases, the SNRs of the linear mode counter output and the average of the LEC output are similar. It is presumed that this was because the SNR in bright illumination is dominated by shot noise and is not affected by variations of the digital gain.

The figure of merit (FoM) defined in [13] was used for comparison with other state-of-the-art sensors. The *FoM* defined in [13] was used for comparison.

(4) $$FoM=\frac{Power\times Noise}{Frame\;rate\times Total\;pixel\;number\times 2^{ADCbit}}$$

Table 2 shows the performance of the proposed CIS and other state-of-the-art technologies. The techniques [7,8,9] refer to the comparison. Except for the proposed CIS, the A-CDS method was used.

## 4. Conclusions

This paper illustrated the concept and structure of an SS-ADC with in-circuit bi-directional gamma correction achieving D-CDS with a hold-and-go counter. The captured images demonstrated that different bi-directional gamma curvatures can be applied by selecting the digital control signal. The measurement results of the prototype sensor demonstrated that the image visibility and the SNR were improved, especially in low-illumination areas. We expect the sensor to have various WDR applications such as computer vision, object detection, etc.

## Figures and Tables

**Table 1:** Measured SNR [dB] with different modes @ 8 lux.

DN @8 lux	Linear	¾ Point B-Gamma	½ Point B-Gamma	¼ Point B-Gamma	Avg. B-Gamma
3.6	11.49	12.79	12.23	12.74	12.69
10.1	18.72	19.19	19.19	19.27	19.22
19.3	22.44	22.73	22.75	22.51	22.66
23.6	23.58	23.81	23.82	23.79	23.81
27.5	24.38	24.58	24.64	24.60	24.61
36.9	25.90	26.08	26.03	25.60	25.90
44.3	26.70	27.06	26.81	26.47	26.78
54.8	27.70	27.87	27.83	27.48	27.73

**Table 2:** Performance comparison; (1) measured with analog gain of 8; (2) input voltage range of ADC is 0.8 V.

	[7]	[8]	[9]	This Work
Types of CDS	A-CDS	A-CDS	A-CDS	D-CDS
Technology [μm]	0.35	0.35	0.11	0.11
Power Dissipation [mW]	N/A	30	6	6.3
Frame Rate	N/A	15 fps	15 fps	16.6 fps
Pixel Size [μm^2^]	9 × 9	5.6 ×5.6	3.2 × 3.2	3.2× 3.2
Random noise [μV_rms_]	N/A	N/A	-	392 ^(1)(2)^
Pixel Array	128 × 128	320 × 240 (QVGA)	640 × 480 (VGA)	640 × 480 (VGA)
Dynamic range [dB]	89	64.8	61.5	61.5
ADC Resolution [bit]	8	10	8	10
FoM [μV·pJ/Conv.step]	N/A	N/A	-	472

## Data Availability

The datasets generated from the current study are available from the corresponding author upon reasonable request.

## References

[1] Huang Z., Zhang X., Chen L., Zhu Y., An F., Wang H., Feng S. (2017). A Vector-Quantization Compression Circuit with On-Chip Learning Ability for High-Speed Image Sensor. IEEE Access.

[2] Bae M., Choi B.S., Jo S.H., Lee H.H., Choi P., Shin J.K. (2016). A Linear-Logarithmic CMOS Image Sensor with Adjustable Dynamic Range. IEEE Sens. J..

[3] Lee S., Yang K. (2006). High dynamic-range CMOS image sensor cell based on self-adaptive photosensing operation. IEEE Trans. Electron Devices.

[4] Shaharom M., Collins S. An integrating wide dynamic range nMOS pixel with a logarithmic reference voltage generator. Proceedings of the 2016 IEEE International Symposium on Circuits and Systems (ISCAS).

[5] Acosta-Serafini P., Masaki I., Sodini C. (2004). A 1/3” VGA linear wide dynamic range CMOS image sensor implementing a predictive multiple sampling algorithm with overlapping integration intervals. IEEE J. Solid-State Circuits.

[6] Ham S., Lee Y., Jung W., Lim S., Yoo K., Chae Y., Han G. CMOS image sensor with analog gamma correction using nonlinear single-slope ADC. Proceedings of the 2006 IEEE International Symposium on Circuits and Systems.

[7] Priyadarshini N., Sarkar M. A High Dynamic Range CMOS Image Sensor using Programmable Linear-Logarithmic Counter for Low Light Imaging Applications. Proceedings of the 2020 IEEE International Symposium on Circuits and Systems (ISCAS).

[8] Kim D., Song M. (2012). An Enhanced Dynamic-Range CMOS Image Sensor Using a Digital Logarithmic Single-Slope ADC. IEEE Trans. Circuits Syst. II Express Briefs.

[9] Im H., Park K., Cho J.H., Choo H.S., Kim S.Y. (2021). Design of a Pseudo-Wide Dynamic Range CMOS Image Sensor by Using the Bidirectional Gamma Curvature Technique. IEEE Trans. Circuits Syst. II Express Briefs.

[10] Baek C., Lim C., Kim D., Song M. Design of a 10-bit CMOS image sensor based on an 8-bit configurable hold-and-go counter. Proceedings of the 2012 ESSCIRC (ESSCIRC).

[11] Park K., Yeom S., Kim S.Y. (2020). Ultra-Low Power CMOS Image Sensor With Two-Step Logical Shift Algorithm-Based Correlated Double Sampling Scheme. IEEE Trans. Circuits Syst. Regul. Pap..

[12] Huang S.C., Cheng F.C., Chiu Y.S. (2013). Efficient Contrast Enhancement Using Adaptive Gamma Correction with Weighting Distribution. IEEE Trans. Image Process..

[13] Park I., Jo W., Park C., Park B., Cheon J., Chae Y. (2020). A 640 × 640 Fully Dynamic CMOS Image Sensor for Always-On Operation. IEEE J. Solid-state Circuits.

